# A Treatise on the Practice of Surgery

**Published:** 1856-11

**Authors:** 


*A Treatise on the Practice of Surgery.* By HENRY H. SMITH, M.D. Professor of the Principles and Practice of Surgery in the University of Pennsylvania; Consulting Surgeon to St. Joseph's Hospital, Philadelphia; Author of a Treatise on Operative Surgery, &c. Illustrated by Two Hundred and Seventy-Four Engravings on Wood. Philadelphia, J. B. Lippencott & Co. 1856.

This is the title to a well-executed volume of eight hundred and twenty-eight pages, recently issued from the press. It was evidently designed by the author to constitute a text-book for the student and a work of reference for the ordinary practitioner. In the preface, the author says:—"Although the title of Practice of Surgery has been taken, the work will be found to contain as full an exposition of the principles of the science as was consistent with its character as a text-book." And, again:—"In the composition of the present Treatise, it has been the author's wish to present each subject as fully as was essential to its comprehension by the youngest of his pupils, without entering into such details of the history, pathology, &c. of each, as properly belong to monographs."

The work is divided into six parts, as follows, viz.:—

*Part I. Surgical Pathology and Therapeutics.*—This embraces three chapters, viz.:—One, on "Surgical Semeiology;" another, on "Diagnosis from an Examination of the External Organs;" and another, on "The Use of the Senses in Forming a Diagnosis."

*Part II. Surgical Pathology of the Soft Tissues.*—This embraces eleven chapters, on the following subjects:—General Characters of Inflammation; Etiology and Treatment of Inflammation; The Effects or Products of Inflammation; Abcesses; Hectic Fever and Pyemia; Ulceration and Ulcers; Mortification; The Specific Forms of Inflammation; Burns; Effects of Cold; and Erysipelas.

*Part III. Pathology of Abnormal Growths in the Soft Tissues.*—This includes only two chapters; one, on Malignant; and the other, on Non-Malignant Growths.

*Part IV. Of Injuries of the Soft Tissues.*—In this Part are included chapters on General Characters of Wounds; on Special Wounds; on Gunshot Wounds; on Tetanus; and on Wounds of the Regions of the Body.

*Part V. Injuries and Diseases of the Bones.*—Under this head are six chapters, viz.:—Of Fractures in General; Fractures of the Head and Face; Fractures of the Neck and Trunk; Fractures of the Upper Extremity; Fractures of the Lower Extremity; Diseases of the Continuity of the Bones.

*Part VI. Injuries and Deseases of the Joints.*—This embraces two chapters, one, on Luxations; and the other, on Luxations of the Bones of the Head and Trunk.

From the foregoing, our readers will readily comprehend the general arrangement and contents of the work. Most of the subjects are considered briefly, perhaps too much so to be satisfactory to the practitioner who wishes for an efficient guide in the treatment of his patients: yet, as a whole, the author has given us a very fair compilation or summary of the doctrines and modes of practice adopted by surgeons of the present day. We use the qualifying term, *as a whole*, because there are some parts that seem to us rather behind the times, and others that scarcely do justice to his contemporaries. For instance, in the section on the "Etiology of Inflammation," the author says:—"The *causes* of inflammation may be classified as exciting, predisposing, and *proximate*." Under this latter head, he proceeds seriously to describe those pathological changes in the blood and tissues which constitue the inflammation itself. In the days of Cullen, when inflammation was defined to be a group of symptoms consisting of heat, redness, pain, and swelling, there was some excuse for applying the term, "Proximate Cause," to the changes in the fluids and solids of the part which gave rise to those symptoms: but, for a systematic writer, in the last half of the nineteenth century, to be guilty of so manifest an absurdity as the application of the term *cause*, or "Proximate Causes," to the disease itself, is scarcely excusable.

Again, in his section on Morbus Coxarius, he fails, in our estimation, to do justice to the valuable paper of Dr. Alden March, of Albany, on the same subject.

In the section on Ununited Fractures, we find the following paragraph, viz.:—"Another method, is that of Dieffenbach, who drilled holes in the extremity of the bones, and inserting little pegs of ivory kept them there, until such an amount of inflammatory action was developed as resulted in union. Dr. Brainard, of Chicago, has also *proposed* to drill a number of holes in the end of the bone with an awl, with the same view. But, in order to make use of any of these modes of treatment, it becomes necessary that the patient should, for a long time, retain the recumbent position, whilst he is exposed to the risks of the creation of a compound fracture, in which suppuration may take place," &c.

This is all the allusion, made by the author, to the highly-interesting and valuable Prize Essay of Dr. Brainard on the subject of Ununited Fractures. The method of simple sub-cutaneous puncture and drilling, adopted by Dr. Brainard, is ranked directly with those methods which involve deep incisions and exposure of more or less of the ends of the bones, thereby leaving an erroneous impression upon the mind of the reader. Again, he speaks of Dr. Brainard as having merely "*proposed* to drill," &c. when he should have said, that Dr. B. *had drilled* or treated by this method numerous cases with entire success.

In the work before us is a section of four pages, on the "Bites of Serpents," in which the author alludes to the experiments performed, many years ago, by "Dr. Barton, formerly Professor of Materia Medica in the University of Pennsylvania, and of Capt. Hall." He also mentions the treatment recommended by Sir David Barry, but makes no allusion whatever to the recent numerous and interesting experiments made by Dr. Brainard, in relation to the efficacy of Iodine as an antidote for the poison of serpents. Such omissions not only do injustice to others, but they greatly lessen the value of the work, even as a text-book, in the hands of students.

There are other things in this work of Dr. Smith open to criticism, did our time and space permit; but we will only add, that the publishers have done their part of the work in good style. The paper and type are fair, the binding substantial, and the cuts or wood engravings numerous and highly-useful. It may be had, we presume, at either of the bookstores in this city where medical books are kept.

